# Draft Genome Sequence of *Elizabethkingia meningoseptica*, Isolated from a Postoperative Endophthalmitis Patient

**DOI:** 10.1128/genomeA.01335-14

**Published:** 2014-12-24

**Authors:** Bulagonda Eswarappa Pradeep, Niranjana Mahalingam, Bhavani Manivannan, Krishnanand Padmanabhan, Pravin Nilawe, Girija Gurung, Amit Chhabra, Valakunja Nagaraja

**Affiliations:** a Department of Biosciences, Sri Sathya Sai Institute of Higher Learning, Prasanthi Nilayam, India; b Department of Microbiology, Sri Sathya Sai Institute of Higher Medical Sciences, Prasanthi Gram, India; c Department of Ophthalmology, Sri Sathya Sai Institute of Higher Medical Sciences, Prasanthi Gram, India; d Cancer Research Program, Rajiv Gandhi Centre for Biotechnology, Thiruvananthapuram, India; e Thermo Fisher Scientific, Mumbai, India; f Department of Microbiology and Cell Biology, Indian Institute of Science, Bengaluru, India

## Abstract

We present the draft genome assembly of an *Elizabethkingia meningoseptica* strain isolated from a 67-year-old postoperative endophthalmitis patient who suffered loss of vision in the right eye. The draft genome assembly has 167 contigs with a total size of 4,019,665 bp encoding multiple drug-resistant genes.

## GENOME ANNOUNCEMENT

*Elizabethkingia meningoseptica*, a Gram-negative ubiquitous bacillus, has been reported to be widely distributed in natural environments (1, 2). It has also been reported to be associated with infections in humans leading to cerebrospinal meningitis in immunocompromised patients and neonates (3, 4), endophthalmitis (5), and keratitis (6). Recently, draft genome sequences of *E. meningoseptica* 502 (7), NBRC 12535^T^, and ATCC 13253^T^ (8) have been reported. Here, we present the draft genome sequence of *E. meningoseptica* isolated from a 67-year-old postoperative endophthalmitis patient who suffered loss of vision in the right eye. The aim of this study was to compare the genome sequence of the test strain with those of recently characterized strains of the species in order to understand the variations in antibiotic resistance.

*E. meningoseptica* was cultured on a MacConkey agar plate and total genomic DNA was extracted from an LB broth culture of a single colony using a genomic DNA extraction kit (Macherey-Nagel, Germany). Antibiotic susceptibility testing of the strain on Vitek-2 exhibited resistance to second and third generation cephalosporins, carbapenems, monobactams, aminoglycosides, but remained susceptible to Levofloxacin and Minocyclin. The genome was sequenced (Invitrogen BioServices India Pvt, Ltd., Gurgaon, India) using an Ion PGM (Personal Genome Machine) sequencing 400 kit and chip 318. Sequencing generated 331,012,462 bases and 1,307,232 reads, with a mean read length of 253 bp. Next, *de novo* assembly was obtained using SPAdes 3.0.0 (9), with *K-mer* values of 21, 33, 55, 77, 99, and 127 assembled into 167 contigs (>200 bp) with *N*_50_ values of 52.39 kb. The total size of the assembly (40,19,665 bp, 82-fold genome coverage) and G+C content (35.49%) are in agreement with the published information for other strains of *E. meningoseptica* (size, 3.96 Mb, 3.84 Mb, and 3.79 Mb; G+C%, 35.85%, 36.2% and 35.2%).

Genome annotation was performed by RAST and NCBI PGAAP. RAST annotation predicted the presence of 4,994 coding sequences and 42 tRNA- and 3 rRNA-encoding genes. Hypothetical genes predicted by RAST were further analyzed using BLASTp and the online Pfam database of protein families. Among the other features, the genes encoding drug efflux/transport systems, resistance to antibiotics (β-lactam, fluoroquinolones), and toxic compounds such as heavy metals were detected. Analysis of unmapped reads revealed the presence of phage genes encoding tail-length tape-measure protein, tail fibers, and endolysin in the test strain, which were not found in the reference strains. The pathogenic and multidrug-resistant features of the strain necessitate further investigations to understand its adaptation as an opportunistic human pathogen. Comparative genomic analysis is being carried out with other *E. meningoseptica* strains to better understand the genetic diversity of the species.

### Nucleotide sequence accession numbers.

The whole-genome shotgun project of this strain has been deposited at DDBJ/EMBL/GenBank under the accession no. JSAA00000000 for *Elizabethkingia meningoseptica* endophthalmitis. The version described in this paper is version JSAA01000000.
