# Correction for Pradeep et al., Draft Genome Sequence of *Elizabethkingia anopheles*, Isolated from a Postoperative Endophthalmitis Patient

**DOI:** 10.1128/genomeA.01372-16

**Published:** 2016-12-08

**Authors:** Bulagonda Eswarappa Pradeep, Niranjana Mahalingam, Bhavani Manivannan, Krishnanand Padmanabhan, Pravin Nilawe, Girija Gurung, Amit Chhabra, Valakunja Nagaraja

**Affiliations:** a Department of Biosciences, Sri Sathya Sai Institute of Higher Learning, Prasanthi Nilayam, India; b Department of Microbiology, Sri Sathya Sai Institute of Higher Medical Sciences, Prasanthi Gram, India; c Department of Ophthalmology, Sri Sathya Sai Institute of Higher Medical Sciences, Prasanthi Gram, India; d Cancer Research Program, Rajiv Gandhi Centre for Biotechnology, Thiruvananthapuram, India; e Thermo Fisher Scientific, Mumbai, India; f Department of Microbiology and Cell Biology, Indian Institute of Science, Bengaluru, India

## AUTHOR CORRECTION

Volume 2, no. 6, e01335-14, 2014. Page 1: The title should read as given above, and strain endophthalmitis should be identified throughout as *Elizabethkingia anopheles* rather than *Elizabethkingia meningoseptica*.

